# Mystery Client Methodologies to Evaluate Abortion Care and Access: A Scoping Review

**DOI:** 10.3390/healthcare14132017

**Published:** 2026-07-07

**Authors:** Martha Paynter, Anja McLeod, Clare Heggie, Alex Goudreau

**Affiliations:** 1 Faculty of Nursing and Health Sciences, University of New Brunswick, Fredericton, NB E3B 5A3, Canada; anja.mcleod@unb.ca; 2 Department of Interdisciplinary Studies, University of New Brunswick, Fredericton, NB E3B 5A3, Canada; clare.heggie@unb.ca; 3 University of New Brunswick Libraries, Saint John, NB E2K 5E2, Canada; alex.goudreau@unb.ca

**Keywords:** mystery client, abortion, mifepristone, access

## Abstract

**Background**: Misinformation, disinformation, and a lack of information about abortion impede access. Mystery shopping designs are well-suited for evaluating the availability, accuracy, and quality of health services. However, we lack understanding of their use in abortion research. **Methods**: Our team conducted a scoping review to synthesize evidence of mystery shopping methods in abortion research internationally. We followed the JBI methodology for scoping reviews and engaged the expertise of a clinical research librarian. We included all English and French language studies of abortion using mystery shopping methods (phone, in person, or other). We did not limit the search by date range or jurisdiction. **Results**: We included 40 studies in our review, published between 2006 and 2025. Settings included 13 countries: USA (13), India (5), Canada (3), Mexico (3), Turkey (3), Bangladesh (2), Colombia (2), Ghana (2), Indonesia (2), and one each in Kenya, Nepal, Tanzania, Zambia, and Latin America. Methods included calls (20), in-person visits (18), texts/online messages (3), and combinations of mystery shopping with interviews/surveys (7) and systematic online searching (2). Themes included service availability and information quality, with seven subthemes, which we mapped to elements of the Levesque framework of patient-centred access: Approachability; acceptability; availability and accommodation; affordability; and appropriateness. **Conclusions**: Where abortion is decriminalized, efforts to improve abortion can prioritize Levesque’s concepts, such as ensuring care is affordable, culturally safe, and geographically proximal. Mystery shopping can proxy patient experience and be used as a validity check, such as comparing health professionals’ self-reported practice with that experienced by mystery shoppers.

## 1. Introduction

While the United States continues to expand legal restrictions on abortion, in recent years, many countries around the world have endeavoured to improve abortion access through decriminalization initiatives. Since 2018, the “Marea Verde”, or Green Wave, has swept Latin America, with countries including Argentina (2020) [1], Colombia (2022) [2], and parts of Mexico (2023) [3] legalizing or partially decriminalizing abortion to enhance gender equity and reduce maternal mortality. The Republic of Ireland made headlines internationally with its 2018 referendum to legalize abortion for up to 12 weeks of pregnancy [4]. Likewise, in 2019, Northern Ireland legalized abortion for all reasons up to week 12 [5]. In 2020, in the early throes of COVID-19, the United Kingdom loosened restrictions on medication abortion to permit telehealth availability and pills-by-mail for pregnancies up to 10 weeks [6]. Worldwide, the pandemic improved provider comfort with telemedicine and expanded access to medication abortion. In 2025, UK lawmakers voted to decriminalize the termination of pregnancy [7]. These are but a handful of examples. Keeping up with rapid changes is challenging for scholars of abortion; for health professionals and the public, this is even more so the case. Without awareness of improvements to abortion care, patients may lack a clear understanding of service pathways and options, and health professionals may lack knowledge about how to appropriately refer and provide care [8]. In this changing context, mystery client methodologies offer an approach to understanding the information available to abortion seekers.

Mystery client study designs are well-suited for evaluating the availability, accuracy, and quality of sexual and reproductive health services [9]. Mystery clients are trained to act as regular patients and collect data about health services from a patient perspective. In the United States (US), mystery client studies have identified inconsistencies in abortion service referral rates and that the accuracy of information provided depends on the restrictiveness of state-level abortion policies and the religious affiliation of the contacted health centres [10,11,12]. Researchers have observed similar patterns in Canadian settings, where organizational type and provincial context influence the information and services available to patients. In the province of Ontario, a 2015 mystery client study of 17 organizations providing postabortion support identified differences in the accuracy of information based on the type of organization (sexual health centre, unregulated crisis pregnancy centre, and both secular and religion-affiliated talk lines) [13]. Guilbert and Bois’ 2021 mystery client study of 50 abortion clinics in Quebec revealed that most of the clinics did not offer medication abortion [14,15]. They found that options for telemedicine were “almost nonexistent”, and the information provided by clinics was insufficient to make informed choices between medication and procedural abortion methods [14,15].

Mystery client methods can be used to assess access to medication abortion through community pharmacies. A 2023 mystery client study of 208 pharmacies in Ontario identified that only 6% had mifepristone in stock when contacted [16]. A 2025 mystery client study of the availability of medication abortion in Alberta found only 12.9% of 1620 responding pharmacies had mifepristone in stock [17]. Mystery client studies can assess the quality and accuracy of information provided to patients by mimicking real-world interactions, while also collecting local and up-to-date information that has direct implications on patient experience and decision making. However, to date, there are no evidence syntheses of mystery client methods in abortion health service research.

Given the need to improve public awareness of advancements in abortion access and the suitability of mystery client methodologies to capture real-world access, our team conducted a scoping review to examine how researchers have used mystery client methods in abortion research internationally. The aim of this scoping review is to synthesize the methods of existing mystery client studies to understand the breadth of use of these approaches. This scoping review addressed studies that used mystery clients in roles as patients, patient partners, or support people, or as health professionals in contexts of family planning services, hospital obstetrics and gynecology departments, unregulated crisis pregnancy centres, pharmacies, health centres, and health insurance providers.

### Theoretical Framework

In this scoping review, we considered the Levesque et al. [18] framework for patient-centred access to healthcare. The framework characterizes access as having five dimensions: approachability (the people’s ability to identify services and reach them); acceptability (cultural and social factors about the service for the care-seeker); availability and accommodation (physical access in a timely manner); affordability; and appropriateness (fit between services and client needs).

## 2. Methods

We followed the JBI methodology for scoping reviews [19] and engaged the expertise of a clinical research librarian at the University of New Brunswick (AG). The most recent search was executed on 20 February 2025.

### 2.1. Inclusion Criteria

We included all studies that used research methods, published in English or French, and that used mystery clients as the method, including information-seeking by phone, in person, or other means. We included all date ranges; importantly, we include both pre- and post-COVID-19 studies. The population for the review includes mystery clients in roles as patients, patient partners, or support people, or as health professionals. We included women and gender-diverse people; youth and adults (excluded: only men). The intervention/exposure includes family-planning services, hospital obstetrics and gynecology departments, unregulated crisis pregnancy centres, pharmacies, health centres, and health insurance providers. The study characteristics include mystery shopping as the primary method in a study or as a part of a multiple-methods study; mystery shopping includes in-person or by telephone, online or text messaging, or other contact methods. An additional consideration for our review is that all studies had to involve some level of deception. While research subjects are not informed of the study, the methodology collects information that employees are authorized to disclose as expected in the normal course of their work.

### 2.2. Exclusion Criteria

We excluded non-research articles, articles not available in English or French, and articles that did not use mystery client methods. We excluded studies not related to abortion care. We excluded studies using known simulated patients for health professional training purposes.

### 2.3. Search Strategy

We designed the search strategy to retrieve both published and unpublished literature, developed by a JBI-trained librarian (AG) following a multi-step process. We conducted an initial, limited search of MEDLINE (Ovid) and Google Scholar to identify relevant publications. Additionally, we identified terms for sexual and reproductive health services from the search strategies of two systematic reviews [20,21]. We used text words from the titles and abstracts of articles from the initial search and reviews, along with corresponding index terms, to create a comprehensive search strategy for MEDLINE. Peers reviewed this strategy according to the Peer Review of Electronic Search Strategies (PRESS) guidelines [22], and we revised the search based on this review.

We adapted the finalized search strategy, including all keywords and index terms, to suit the syntax and controlled vocabulary of the following databases: MEDLINE (Ovid) (see Table S1), CINAHL with Full Text (EBSCOhost) (see Table S2), Academic Search Premier (EBSCOhost), Women’s Studies International (EBSCOhost) (see Table S3), Web of Science Core Collection (Clarivate) (see Table S4), and Embase (Elsevier) (see Table S5). We searched all databases from inception to 20 February 2025, without applying any restrictions. Full search strategies for each database are detailed in [Supplementary Material] and are available in a data repository [23,24,25].

We designed the database search strategies as presented for the original, broader research question, which intended to capture all aspects of sexual and reproductive health services (*How have mystery client methods been used to examine access to reproductive health services?*), including abortion and related abortion medication. However, many of the results did not relate to abortion. After consultation with the clinical librarian, the team determined it to be unnecessary to revise and rerun the search strategies as they already included all necessary terms for the new focus. The eligibility criteria for included studies were revised to exclude studies that focused on reproductive health, not including abortion. Examples included: PReP; ART compliance; Teratogenic medicines; STI screening or treatment; Prenatal care; and OUD/OAT in pregnancy.

We manually screened the reference lists of all included studies for further eligible studies and performed forward citation tracking using Google Scholar and Scopus (Elsevier).

### 2.4. Study/Source of Evidence Selection

We collected and uploaded all identified citations from the database searches into Covidence systematic review software (2026, Veritas Health Innovation, Melbourne, Australia). Covidence automatically identified and removed duplicates.

### 2.5. Study Selection

**Figure 1:**
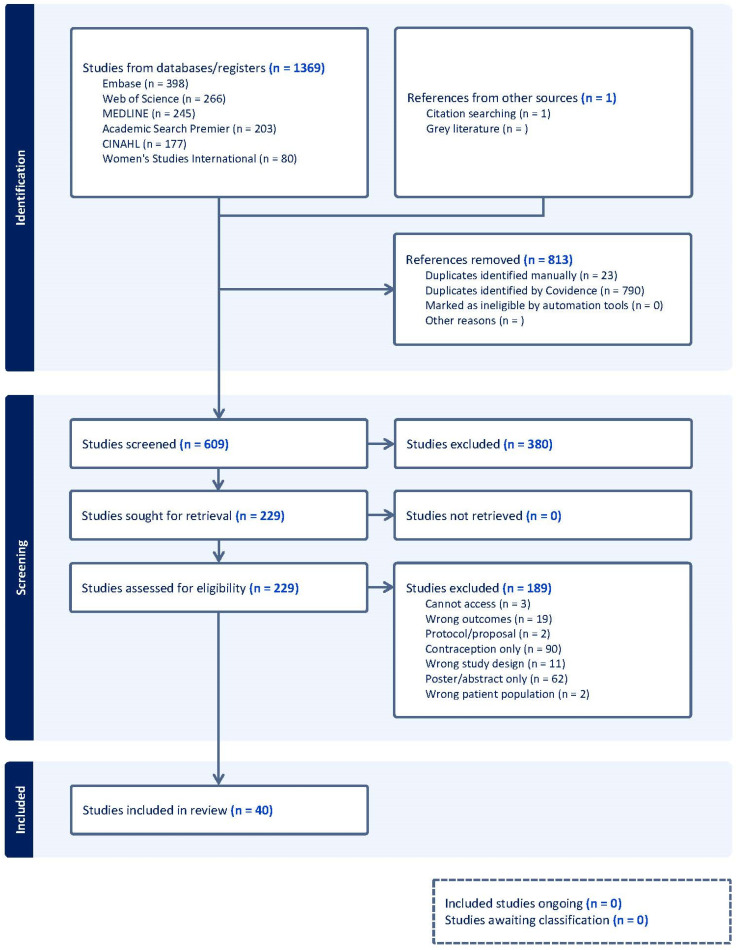
PRISMA diagram.

Three independent reviewers (MP, CH, and AM) screened all titles and abstracts for assessment against the inclusion criteria for the review. We retrieved potentially relevant sources in full and imported their citation details into Covidence. Two independent reviewers (LT, AM) assessed the full text of selected citations in detail against the inclusion criteria. We recorded reasons for exclusion of sources of evidence in the full text. We resolved any disagreements between the reviewers at each stage of the selection process through discussion or with an additional member of the author team. The results of the search and the study-inclusion process are presented in a Preferred Reporting Items for Systematic Reviews and Meta-analyses extension for scoping review (PRISMA-ScR) flow diagram [26]. See Figure 1.

### 2.6. Data Extraction

Two independent reviewers (LT and AM) extracted data from papers included in the scoping review using a data extraction tool developed by our team. The authors have worked together on several scoping reviews, and the data extraction tool from our previous scoping reviews was replicated and specified for the purpose of this study. Reviewers extracted data independently on five articles as a pilot test and compared the extraction results. Any discrepancies were resolved through discussion with the full research team (MP, CH, AM, and LT). The data extracted included author, year, jurisdiction (country), study design (calls, in-person visit, text/online message, or other), type of participants (patient, healthcare provider, partner of patient, or other), setting (type of facility/health professional being “shopped”), number and type of “profiles” of “shoppers”, shopper training information, sample size (number of the facilities/professionals being shopped), outcomes of interest, and relevant key findings. We then grouped studies into relevant types and themes. Articles were first categorized based on the main themes reflecting the dominant focus of the article, then further organized into subthemes according to specific issues or outcomes of interest. The Levesque conceptual framework for access to healthcare was then used to map study outcomes onto aspects of access [18].

## 3. Results

### 3.1. Study Characteristics

We included 40 studies, published between 2006 and 2025 (see Table S6). Study jurisdictions included 13 countries, including: USA (13) [27,28,29,30,31,32,33,34,35,36,37,38,39], India (5) [40,41,42,43,44], Canada (3) [14,15,16], Mexico (3) [45,46,47], Turkey (3) [48,49,50], Bangladesh (2) [51,52], Colombia (2) [53,54], Ghana (2) [55,56], Indonesia (2) [57,58], and one study each in Kenya [59], Nepal [60], Tanzania [61], Zambia [62], and Latin America generally [63].

Mystery client data collection methods included calls, in-person visits, and texts/online messages. Some studies combined these methods and/or used these methods and systematic online searching or interviews/surveys. Twenty (20) studies used mystery client phone calls: 14 used calls only [14,15,16,28,31,33,34,35,36,37,39,48,49,50], two (2) used calls and monthly service data from abortion clinics [27,38], two (2) used calls and systematic online searches [30,32], one used calls and a survey [41], and one used calls and visits [29]. Eighteen studies, including the latter, used in-person visits by mystery clients. Eleven (11) used visits only [42,43,45,47,51,54,56,59,60,61,62], three (3) used visits and interviews [44,52,63], two (2) used visits and surveys [46,55], and one (1) used visits, surveys, and interviews [40]. Three studies used mystery client texts or online messaging [53,57,58].

In two (2) studies, the mystery clients presented as a physician or medical student looking for mifepristone for a patient [16,28]. Two (2) other studies used a mystery client calling on behalf of a patient seeking abortion [32,34]. Fourteen (14) studies used a profile of a young female abortion-seeker between the ages of 17 and 35 [14,15,31,39,45,48,49,50,53,54,56,57,58,59]. One study used the mystery client profile of a youth abortion-seeker aged 12–17 [46]. Thirteen (13) studies used a combination of both a profile of a young female abortion-seeker and of a male partner of a pregnant person [29,40,41,42,43,44,47,51,52,55,60,62,63]. In eight (8) studies, the profile of the mystery client was not specified [27,30,33,35,36,37,38,61].

Clinical settings and/or staff being studied included abortion clinics (10) [14,15,27,30,32,33,36,37,38,46], unregulated/crisis pregnancy centres (2) [29,31], both abortion clinics and unregulated/crisis pregnancy centres (1) [39], abortion care providers (1) [56], obstetrics department staff (3) [48,49,50], online abortion pill sellers (3) [53,57,58], United States Medicaid (1) [34], and pharmacy staff (19) [16,28,35,40,41,42,43,44,45,47,51,52,54,55,59,60,61,62,63]. Twenty-seven studies focused exclusively on medication abortion (MA) [14,16,28,33,35,36,37,40,41,42,43,44,45,47,48,51,52,53,54,55,57,58,59,60,61,62,63].

Sample sizes ranged from three abortion clinics [27] to 977 hospitals [50]. Studies used between one (1) and four (4) mystery client “profiles”. Studies used between one (1) and 48 individuals to conduct up to 576 [47] in-person visits and 1954 calls [49]. The most commonly reported training approach for mystery clients involved extensive rehearsal and role-play [43,44,51,52,59,60] and adherence to a standardized script [31,33,34,44,49,50]. Some studies stated that mystery clients received training but provided no further details [14,15,53], while others reported mystery clients had extensive experience as mystery clients or in related research [42,45,48,54]. Two (2) studies held one-day training sessions [56,57], three (3) reported two-day training [31,46,59], and four (4) followed a three-day training process [40,41,43,60]. Of the studies that trained mystery clients for three days, three (3) incorporated pilot testing of their procedures in the field at non-participating locations [40,43,60]. The remaining fifteen studies did not report details regarding mystery client training [16,27,28,29,30,32,35,36,37,38,39,47,61,62,63].

Our analysis found two overarching themes and seven subthemes, which we mapped to four of Levesque’s five access concepts. See Table 1.

### 3.2. Service Availability

Thirty-two (32) of the 40 studies evaluated the availability of abortion services [14,16,27,28,30,32,33,34,35,36,37,38,39,42,44,45,47,48,49,50,51,52,54,55,56,57,58,59,60,61,62,63]. Twenty-three (23) of these focused exclusively on the availability of MA [14,16,28,32,33,35,36,37,42,44,45,47,51,52,54,55,57,58,59,60,61,62,63], three (3) on procedural abortion services only [48,49,50], five (5) examined abortion services broadly or across methods [27,30,34,38,39], and one (1) explored second-trimester abortion service availability only [56].

### 3.3. Stocking Medication Abortion

Ten (10) studies investigated whether a pharmacy had MA medication in stock [16,28,35,42,44,45,47,54,55,63], and in three (3) of these studies, mystery clients asked whether the medication could be ordered if not in stock [16,28,35]. One of these also asked pharmacy staff the reasons for medication not being in stock [16]. Six (6) studies analyzed the referral pathways suggested by pharmacy staff if they did not stock MA [45,52,54,55,60,62]. Two (2) studies assessed the likelihood of receiving MA along gender lines, i.e., they compared the likelihood of pharmacy staff to dispense MA to male mystery clients posing as partners compared to female mystery clients posing as abortion-seekers [51,60].

### 3.4. Wait Times

Seven (7) studies sought information on wait times for abortion care [14,28,35,36,37,38,39], one of which specifically examined wait time for medication abortion [35].

### 3.5. Costs

Seven (7) studies collected information about the cost of abortion services [34,36,37,39,45,50,56]; two (2) of these examined health insurance coverage in the United States (US) [34,36].

### 3.6. Geographic Distance

Five (5) studies measured geographic distance to care or geographic distribution of services [14,27,30,36,37]. Four (4) of these used mystery clients to determine the operational status of clinics and providers, and then calculated travel distance or time from a defined origin, such as university and college campuses [36,37], or cities with populations of 50,000 or more [30], to the nearest clinic among those currently in operation, and one combined mystery client methodology with an analysis of service data to measure the actual distances travelled by patients [27].

### 3.7. COVID-19/Telehealth

Three (3) studies, all based in the US and using call methods, investigated service availability during the COVID-19 pandemic [27,32,38]. Roberts et al. determined the proportion of clinics operating during the pandemic, the proportion offering MA versus PA, and the median and range of wait times [38]. Berglas et al. determined the operational status of the clinics at the pandemic onset [27]. Kaller et al. collected information on abortion facilities’ COVID-19 protocols and documented any telehealth options offered to the mystery clients [32].

### 3.8. Information Quality

Twenty-five (25) studies examined the quality of information provided to mystery clients, including subthemes of clinical accuracy and professional attitudes [14,15,29,31,39,40,41,42,43,44,45,46,47,48,51,52,53,54,57,58,59,60,61,62,63].

### 3.9. Clinical Accuracy

Ten (10) studies assessed the counselling provided by pharmacy staff regarding MA, including dosage, administration instructions, side effects, follow-up care, and potential complications [43,44,45,47,51,52,54,59,60,63].

One (1) study assessed the adequacy of the information provided by abortion clinics to clients to make an informed choice between MA and PA [15].

Two (2) studies made calls to unregulated crisis pregnancy centres (CPCs) to assess the accuracy of medical information patients seeking abortion receive when encountering these centres [29,31].

Seven (7) studies compared provider responses in surveys or interviews with mystery client findings [40,41,43,44,46,52,63].

Three (3) studies evaluated information from online sellers of MA [53,57,58]. In these three studies, mystery clients made contact by text or online messaging, purchased the medication, and, upon receipt, examined the contents of the packages they received by mail [53,57,58].

### 3.10. Professional Attitude

In six (6) studies [14,15,42,43,46,62], mystery clients evaluated the quality of the interaction with healthcare professionals during calls or in-person visits. Dixit et al. assessed pharmacists’ attitudes as neutral, negative, or positive [42]. Percher et al. invited mystery clients to provide additional notes about their interactions with pharmacy staff, including staff “tone” [43]. Guilbert and Bois evaluated abortion clinic staff attitudes on a scale from 1 (unpleasant) to 9 (friendly) [14,15]. Clyde et al. interviewed mystery clients immediately after they conducted in-person visits, asking about the visit flow, treatment by staff, and staff reactions to the request for abortion services [46]. Mystery clients in Hendrickson et al.’s study recorded whether they would describe their interactions with pharmacy staff as “hostile” [62].

Five (5) studies evaluated the information provided to mystery clients according to attributes of the mystery client, such as whether a male partner or female patient sought the abortion information, or a married abortion-seeker vs. unmarried [40,43,46,51,60]. Clyde et al. evaluated the differences in information provided to adolescent patients when seeking care alone versus when accompanied by an adult [46].

## 4. Discussion

In this scoping review, we sought to synthesize the types of mystery client methods used internationally in studies about abortion services. Among the 40 included studies, we found mystery shopping telephone calls to be the most used method, followed by in-person visits, and a small number of studies used text/online messages. It is unsurprising that calls would dominate, as this method is low-cost and potentially the most logistically feasible. Calls offer much stronger anonymity compared to in-person visits, which may present more ethical concerns or potential personal risks. We would expect use of messaging approaches to increase in the future, as this method becomes more commonplace in clinical settings. Common methodological limitations of mystery client studies for consideration include the deception required, although the information collected would be expected in the normal course of a pharmacist’s or clinician’s work. Additional limitations include potential variability among practitioners at the same pharmacy or clinic location; for example, the answers provided by one pharmacist may not be reflective of all those working at the same site [16].

Over one-quarter of the studies (12) used mystery client approaches in combination with other methods, including comparing results when a mystery client seeks information to when a health professional self-reports on their practices. These approaches serve as a useful validity check, can expose intrinsic health professional biases, and illuminate the limitations of self-report methods to understand de facto practices. For example, Diamond-Smith et al. found mystery clients reported lower-quality and less accurate information compared to the information provided by pharmacists who self-reported [40]. Powell-Jackson et al. determined that pharmacists self-reported strong knowledge of MA, such as warning signs to seek emergent care, but did not offer this information to mystery clients [44]. Through mystery client visits, Percher et al. found that more pharmacies sold misoprostol than pharmacists reported by survey [43]. Lara et al. found pharmacy staff offered MA information to mystery clients in person, despite low rates of self-reported knowledge about MA in a survey conducted among the same pharmacy locations. This is a promising use of mystery client methods [63].

Two overarching themes emerged in our scoping review: Service availability and information quality. At its most basic, a mystery client can verify if care exists where they are, or not; if a clinic is operational or not; if medication abortion is in stock or not; and if telehealth is an option or not. These questions map onto the Levesque concept of “availability and accommodation” [18].

Studies from a wide range of countries and settings were included, with considerable variation in the legal, policy, and healthcare delivery contexts. The high methodological heterogeneity observed across the studies suggests the adaptability of mystery client methodologies to diverse contexts. For example, mystery client methodologies may be particularly useful in jurisdictions with restricted abortion access, as they offer insight into services that are practically available. Contrastingly, studies conducted following legislative or policy changes may be positioned to examine service uptake and accessibility, while studies in protective environments such as Canada can serve to evaluate the quality of care being provided.

Notably, nearly one-third (13) of the studies in our review are set in the US, with most of these (8) published after the Dobbs decision of June 2022. As of writing, 41 US states now have some type of abortion ban, with high variability between them and persistent threats of further restrictions [64]. Mystery shopping is likely to continue to serve as an important method to evaluate shifts in service availability.

Beyond the question of availability, this scoping review demonstrates how mystery client methods can examine other dimensions of “access” identified by Levesque: acceptability, affordability, and appropriateness [18]. In jurisdictions less burdened by legal restrictions, efforts to improve abortion services can focus on ensuring care is affordable, culturally safe, non-judgmental, and evidence-based. Future mystery client studies in abortion care could further interrogate equity considerations and illuminate the characteristics of care provided to systemically underserved patient groups, such as members of the 2SLGTBQIA+ communities, Indigenous, Black and racialized peoples, and people with disabilities.

### Limitations

Our review has several limitations. We only included articles available in English or French and may have excluded important research published in other languages. Although assessment of methodological quality is not a requirement of scoping reviews, not assessing for risk of bias is acknowledged as a possible limitation to scoping review methods. Despite using a systematic approach to conduct a comprehensive and broad search strategy, some relevant research studies may have been missed.

## 5. Conclusions

This scoping review aimed to synthesize mystery client methods used in abortion services research. The predominant methods among the studies include mystery client calls, visits, and text/online messaging, with mystery shopping also used to complement other methods. The studies can be grouped into two types: studies of service availability and of information quality. The results of this review demonstrate the utility of mystery shop approaches to proxy patient experience and investigate broader concepts of access beyond legality and availability. In the future, researchers may use mystery shopping to investigate health equity considerations such as differential treatment among underserved patient groups.

## Figures and Tables

**Table 1:** Themes, subthemes, and Levesque’s access concept among included articles.

Main Theme	Subthemes	Levesque Access Concept
Service availability (32)	Stocking medication abortion	Availability/accommodation
	Wait Times	Availability/accommodation
	Geographic distance	Availability/accommodation
	Cost	Affordability
	COVID-19/Telehealth	Availability/accommodation
Information quality (24)	Clinical accuracy	Appropriateness
	Professional attitude	Acceptability

## Data Availability

No new data were created or analyzed in this study.

## Supplementary Materials

The following supporting information can be downloaded at: https://www.mdpi.com/article/10.3390/healthcare14132017/s1, Table S1: Search Strategies from Ovid MEDLINE(R) and Epub Ahead of Print; Table S2: Search Strategies from CINAHL database; Table S3: Search Strategies from Women’s Studies International; Table S4: Search Strategies from Web of Science Core Collection (Clarivate); Table S5: Search Strategies from EMBASE (Elsevier); Table S6: Mystery client methodologies to evaluate abortion care and access.
