# Supplementary material for: Mystery Client Methodologies to Evaluate Abortion Care and Access: A Scoping Review
Source: Healthcare (Basel). 2026 Jul 7;14(13):2017. doi: 10.3390/healthcare14132017 (PMC13361521; doi:10.3390/healthcare14132017)
Supplement: Supplementary file 1 [file healthcare-14-02017-s001.zip › healthcare-4350830-supplementary.pdf]

Supplementary Materials

Search Strategies

The following search strategies are presented exactly as they appeared in the search histories for each database. Each database displays search histories differently, and PRISMA-Searching reporting guidelines recommend copying and pasting the history as run with no edits [24]. For some databases like those on EBSCOhost platforms, this means the search must be read upwards rather than downwards as is presented with Medline search histories.

These search strategies and additional information about the search creation process is available in the University of New Brunswick's data repository [23,25].

Database: Ovid MEDLINE(R) and Epub Ahead of Print, In-Process, In-Data-Review & Other Non-Indexed Citations, Daily and Versions < 1946 to February 19, 2025 >; searched February 20, 2025

Table S1. Search Strategies from Ovid MEDLINE(R) and Epub Ahead of Print

#	Query	Results
1	(Mystery adj (client* or shopp* or customer* or patient*)).ab,kf,kw,ti.	336
2	(secret adj (client* or shopp* or customer* or patient*)).ab,kf,kw,ti.	148
3	(covert* adj (client* or shopp* or customer* or patient*)).ab,kf,kw,ti.	14
4	(undercover adj (client* or shopp* or customer* or patient*)).ab,kf,kw,ti.	4
5	(Unannounce* adj3 patient*).ab,kf,kw,ti.	137
6	(unannounce* adj (client* or shopp* or customer* or patient* or actor*)).ab,kf,kw,ti.	13
7	(Incognito adj (client* or shopp* or customer*)).ab,kf,kw,ti.	1
8	(Incognito adj2 patient*).ab,kf,kw,ti.	27
9	simulat* client*.ab,kf,kw,ti.	187
10	exp Reproductive Health Services/	46,068
11	exp Women's Health Services/	6797
12	exp Contraceptive Agents/	80,697
13	exp Contraception/	30,601
14	exp Contraceptive Devices/	27,635
15	exp Obstetric Surgical Procedures/	155,053
16	Menstruation/	16,810
17	exp Abortifacient Agents/	66,483
18	Misoprostol/	4774
19	Mifepristone/	6627

20	exp Pregnancy/	1,052,387
21	Maternal Health/	2707
22	exp Reproductive Behavior/	10,720
23	Reproductive Health/	5921
24	exp Fertility/	48,607
25	exp Fertility Agents/	30,863
26	exp HIV Infections/	328,227
27	exp Sexual Behavior/	130,946
28	Sexual Health/	3253
29	Gynecological Examination/	789
30	exp Genital Diseases/	1,157,773
31	exp Reproductive Physiological Phenomena/	1,622,940
32	"Contracept*".ab,kf,kw,ti.	84,832
33	"abortion*".ab,kf,kw,ti.	73,837
34	"menstrua*".ab,kf,kw,ti.	62,193
35	"pregnan*".ab,kf,kw,ti.	659,982
36	(family adj2 plan*).ab,kf,kw,ti.	50,192
37	birth control.ab,kf,kw,ti.	5906
38	(Sexually transmitted adj3 infection*).ab,kf,kw,ti.	21,965
39	Acquired Immunodeficiency Syndrome.ab,kf,kw,ti.	21,275
40	HIV.ab,kf,kw,ti.	376,208
41	"Sexually Transmitted Disease*".ab,kf,kw,ti.	19,122
42	"gyn?ecolog* exam*".ab,kf,kw,ti.	2793
43	fertility.ab,kf,kw,ti.	117,659
44	(matern* adj3 (service* or care or healthcare)).ab,kf,kw,ti.	23,468
45	(wom?n adj3 (service* or care or healthcare)).ab,kf,kw,ti.	27,670
46	(sexual* adj3 (service* or care or healthcare)).ab,kf,kw,ti.	6000
47	(reproduct* adj3 (service* or care or healthcare)).ab,kf,kw,ti.	7266
48	1 or 2 or 3 or 4 or 5 or 6 or 7 or 8 or 9	837
49	10 or 11 or 12 or 13 or 14 or 15 or 16 or 17 or 18 or 19 or 20 or 21 or 22 or 23 or 24 or 25 or 26 or 27 or 28 or 29 or 30 or 31 or 32 or 33 or 34 or 35 or 36 or 37 or 38 or 39 or 40 or 41 or 42 or 43 or 44 or 45 or 46 or 47	3,119,748
50	48 and 49	245

Database: CINAHL with Full-Text (EBSCOhost); searched February 20, 2025

Database details from search history:

- Limiters/Expanders: Expanders - Apply related words; Apply equivalent subjects
  Search modes - Find all my search terms
- Last Run Via: Interface - EBSCOhost Research Databases; Search Screen - Advanced Search; Database - CINAHL with Full Text

Table S2. Search Strategies from CINAHL database

#	Query	Results
S51	S49 AND S50	177
S50	S9 OR S10 OR S11 OR S12 OR S13 OR S14 OR S15 OR S16 OR S17 OR S18 OR S19 OR S20 OR S21 OR S22 OR S23 OR S24 OR S25 OR S26 OR S27 OR S28 OR S29 OR S30 OR S31 OR S32 OR S33 OR S34 OR S35 OR S36 OR S37 OR S38 OR S39 OR S40 OR S41 OR S42 OR S43 OR S44 OR S45 OR S46 OR S47 OR S48	905,437
S49	S1 OR S2 OR S3 OR S4 OR S5 OR S6 OR S7 OR S8	479
S48	TI HIV OR AB HIV	111,602
S47	TI "Acquired Immunodeficiency Syndrome" OR AB Acquired Immunodeficiency Syndrome	3447
S46	TI "Sexually Transmitted Disease*" OR AB "Sexually Transmitted Disease*"	5128
S45	TI Sexually transmitted N3 infection* OR AB Sexually transmitted N3 infection*	9735
S44	TI "gyn?ecolog* exam*" OR AB "gyn?ecolog* exam*"	128
S43	TI fertility OR AB fertility	16103
S42	TI "birth control" OR AB "birth control"	1714
S41	TI family N2 plan* OR AB family N2 plan*	8863
S40	TI menstrua* OR AB menstrua*	13,016
S39	TI abortion* OR AB abortion*	14,173
S38	TI pregnan* OR AB pregnan*	175,158
S37	TI Contracept* OR AB Contracept*	23,794
S36	TI (matern* N3 (service* or care or healthcare)) OR AB (matern* N3 (service* or care or healthcare))	14,363
S35	TI (wom?n* N3 (service* or care or healthcare)) OR AB (wom?n* N3 (service* or care or healthcare))	27,498
S34	TI (sexual* N3 (service* or care or healthcare)) OR AB (sexual* N3 (service* or care or healthcare))	5713
S33	TI (reproduct* N3 (service* or care or healthcare)) OR AB (reproduct* N3 (service* or care or healthcare))	4188
S32	(MH "Mammography")	13,757
S31	(MH "Sexual Assault Examination")	198
S30	(MH "Sexual Health Clinics")	38
S29	(MH "Obstetric Service")	1416
S28	(MH "Midwifery Service+")	2139
S27	(MH "Fertility Clinics")	147
S26	(MH "Sexual Behavior+")	55,527
S25	(MH "Sexually Transmitted Diseases+")	117,723

S24	(MH "Surgery, Ob-Gyn+")	55,618
S23	(MH "Diagnosis, Ob-Gyn+")	40,299
S22	(MH "Reproductive and Urinary Physiology+")	338,519
S21	(MH "Female Urogenital Diseases and Pregnancy Complications+")	381,992
S20	(MH "Reproductive Control Agents+")	32,474
S19	(MH "Contraceptive Devices+")	13,449
S18	(MH "Contraception+")	12,741
S17	(MH "Family Planning")	7806
S16	(MH "Women's Health")	50,583
S15	(MH "Sexual Health")	9650
S14	(MH "Reproductive Health")	11,127
S13	(MH "Family Health")	6542
S12	(MH "Maternal Health Services+")	38,884
S11	(MH "Maternal-Child Health")	4477
S10	(MH "Women's Health Services")	2785
S9	(MH "Sexual Health Services")	113
S8	TI simulat* client* OR AB simulat* client*	506
S7	TI Incognito N2 patient* OR AB Incognito N2 patient*	18
S6	TI Unannounce* N3 patient* OR AB Unannounce* N3 patient*	79
S5	TI (unannounce* N1 (client* or shopp* or customer* or patient* or actor*)) OR AB (unannounce* N1 (client* or shopp* or customer* or patient*or actor*))	44
S4	TI (undercover N1 (client* or shopp* or customer* or patient*)) OR AB (undercover N1 (client* or shopp* or customer* or patient*))	7
S3	TI (covert* N1 (client* or shopp* or customer* or patient*)) OR AB (covert* N1 (client* or shopp* or customer* or patient*))	46
S2	TI (secret N1 (client* or shopp* or customer* or patient*)) OR AB (secret N1 (client* or shopp* or customer* or patient*))	131
S1	TI (Mystery N1 (client* or shopp* or customer* or patient*)) OR AB (Mystery N1 (client* or shopp* or customer* or patient*))	209

Databases: Academic Search; Women's Studies International (EBSCOhost) – searched February 20, 2025

Database details from search history:

- Limiters/Expanders: Expanders - Apply related words; Apply equivalent subjects
  Search modes - Find all my search terms
- Last Run Via: Interface - EBSCOhost Research Databases; Search Screen - Advanced Search; Database- Academic Search Premier; Women's Studies International

Table S3. Search Strategies from Women's Studies International

#	Query	Results
S27	S25 AND S26	283
S26	S9 OR S10 OR S11 OR S12 OR S13 OR S14 OR S15 OR S16 OR S17 OR S18 OR S19 OR S20 OR S21 OR S22 OR S23 OR S24	933,390
S25	S1 OR S2 OR S3 OR S4 OR S5 OR S6 OR S7 OR S8	3080
S24	TI HIV OR AB HIV	240,017
S23	TI "Acquired Immunodeficiency Syndrome" OR AB Acquired Immunodeficiency Syndrome	9846
S22	TI "Sexually Transmitted Disease*" OR AB "Sexually Transmitted Disease*"	15,798
S21	TI Sexually transmitted N3 infection* OR AB Sexually transmitted N3 infection*	16,773
S20	TI "gyn?ecolog* exam*" OR AB "gyn?ecolog* exam*"	310
S19	TI fertility OR AB fertility	105,357
S18	TI "birth control" OR AB "birth control"	10,634
S17	TI family N2 plan* OR AB family N2 plan*	41,610
S16	TI menstrua* OR AB menstrua*	38,858
S15	TI abortion* OR AB abortion*	92,150
S14	TI pregnan* OR AB pregnan*	406,990
S13	TI Contracept* OR AB Contracept*	72,437
S12	TI (matern* N3 (service* or care or healthcare)) OR AB (matern* N3 (service* or care or healthcare))	19,887
S11	TI (wom?n* N3 (service* or care or healthcare)) OR AB (wom?n* N3 (service* or care or healthcare))	49,692

S10	TI (sexual* N3 (service* or care or healthcare)) OR AB (sexual* N3 (service* or care or healthcare))	9246
S9	TI (reproduct* N3 (service* or care or healthcare)) OR AB (reproduct* N3 (service* or care or healthcare))	9844
S8	TI simulat* client* OR AB simulat* client*	2144
S7	TI Incognito N2 patient* OR AB Incognito N2 patient*	24
S6	TI Unannounce* N3 patient* OR AB Unannounce* N3 patient*	94
S5	TI (unannounce* N1 (client* or shopp* or customer* or patient* or actor*)) OR AB (unannounce* N1 (client* or shopp* or customer* or patient*or actor*))	34
S4	TI (undercover N1 (client* or shopp* or customer* or patient*)) OR AB (undercover N1 (client* or shopp* or customer* or patient*))	21
S3	TI (covert* N1 (client* or shopp* or customer* or patient*)) OR AB (covert* N1 (client* or shopp* or customer* or patient*))	64
S2	TI (secret N1 (client* or shopp* or customer* or patient*)) OR AB (secret N1 (client* or shopp* or customer* or patient*))	319
S1	TI (Mystery N1 (client* or shopp* or customer* or patient*)) OR AB (Mystery N1 (client* or shopp* or customer* or patient*))	446

Database: Web of Science Core Collection (Clarivate); searched on February 20, 2025

Table S4. Search Strategies from Web of Science Core Collection (Clarivate)

#	Search Query	Results
1	TS=(Mystery NEAR (client* or shopp* or customer* or patient*))	848
2	TS=(secret NEAR (client* or shopp* or customer* or patient*))	1142
3	TS=(covert* NEAR (client* or shopp* or customer* or patient*))	1075
4	TS=(unannounce* NEAR (client* or shopp* or customer* or patient* or actor*))	284
5	TS=(Unannounce* NEAR/3 patient*)	197
6	TS=(Incognito NEAR (client* or shopp* or customer*))	7
7	TS=(Incognito NEAR/2 patient*)	36
8	TS=(reproduct* NEAR/3 (service* or care or healthcare))	10643
9	TS=(sexual* NEAR/3 (service* or care or healthcare))	10542
10	TS=(wom?n NEAR/3 (service* or care or healthcare))	45072
11	TS=(matern* NEAR/3 (service* or care or healthcare))	28259
12	TS=pregnan*	744432
13	TS=Contracept*	83513
14	TS=aborti*	89966
15	TS=(family NEAR/2 plan*)	41873
16	TS="birth control"	6044
17	TS=fertility	203187
18	TS=Misoprostol	8092
19	TS=Mifepristone	5986
20	TS=menstrua*	70891
21	TS="gyn?ecolog* exam*"	508
22	TS=Sexually transmitted infection*	36234
23	TS="Sexually Transmitted Disease*"	20253
24	TS="Acquired Immunodeficiency Syndrome"	26209
25	TS=HIV	464643
26	TS="human immunodeficiency virus infection"	7883
27	#26 OR #25 OR #24 OR #23 OR #22 OR #21 OR #20 OR #19 OR #18 OR #17 OR #16 OR #15 OR #14 OR #13 OR #12 OR #11 OR #10 OR #9 OR #8	1621468
28	#7 OR #6 OR #5 OR #4 OR #3 OR #2 OR #1	3338
29	#28 AND #27	266

Database: EMBASE (Elsevier); searched on February 20, 2025

Table S5. Search Strategies from EMBASE (Elsevier)

No.	Query	Results
#46	#44 AND #45	398
#45	#9 OR #10 OR #11 OR #12 OR #13 OR #14 OR #15 OR #16 OR #17 OR #18 OR #19 OR #20 OR #21 OR #22 OR #23 OR #24 OR #25 OR #26 OR #27 OR #28 OR #29 OR #30 OR #31 OR #32 OR #33 OR #34 OR #35 OR #36 OR #37 OR #38 OR #39 OR #40 OR #41 OR #42 OR #43	5733764
#44	#1 OR #2 OR #3 OR #4 OR #5 OR #6 OR #7 OR #8	1217
#43	(matern* NEAR/3 (service* OR care OR healthcare)):ti,ab,kw	25439
#42	(wom?n* NEAR/3 (service* OR care OR healthcare)):ti,ab,kw	39416
#41	(sexual* NEAR/3 (service* OR care OR healthcare)):ti,ab,kw	8561
#40	(reproduct* NEAR/3 (service* OR care OR healthcare)):ti,ab,kw	9995
#39	fertility:ti,ab,kw	141372
#38	'gyn?ecolog* exam*':ti,ab,kw	996
#37	'sexually transmitted disease*':ti,ab,kw	22138
#36	hiv:ti,ab,kw	488411
#35	'acquired immunodeficiency syndrome':ti,ab,kw	20337
#34	('sexually transmitted' NEAR/3 infection*):ti,ab,kw	28469
#33	'birth control':ti,ab,kw	6209
#32	(family NEAR/2 plan*):ti,ab,kw	31040
#31	pregnan*:ti,ab,kw	879058
#30	menstrua*:ti,ab,kw	82798
#29	abortion*:ti,ab,kw	87367
#28	contracept*:ti,ab,kw	101691
#27	'human immunodeficiency virus infection'/exp	847590
#26	'genital system disease'/exp	1670587
#25	'sexual behavior'/exp	276166
#24	'genital system examination'/exp	193132
#23	'gynecologic surgery'/exp	207529
#22	'obstetric procedure'/exp	639377
#21	'genital system function'/exp	552792
#20	'abortion'/exp	110158
#19	'reproduction'/exp	1596379
#18	'contraceptive device'/exp	53499
#17	'agents acting on the genital system'/exp	1185247
#16	'contraception'/exp	203888
#15	'family planning'/exp	45499
#14	'family health'/exp	12116
#13	'reproductive health'/exp	27566
#12	'women`s health'/exp	35981
#11	'sexual health'/exp	25401
#10	'family service'/exp	772
#9	'maternal health service'/exp	3550
#8	'simulat* client*':ti,ab,kw	219
#7	(incognito NEAR/2 patient*):ti,ab,kw	34

#6	(unannounce* NEAR/3 patient*):ti,ab,kw	209
#5	(unannounce* NEAR/1 (client* OR shopp* OR customer* OR patient* OR actor*)):ti,ab,kw	13
#4	(undercover NEAR/1 (client* OR shopp* OR customer* OR patient*)):ti,ab,kw	8
#3	(covert* NEAR/1 (client* OR shopp* OR customer* OR patient*)):ti,ab,kw	27
#2	(secret NEAR/1 (client* OR shopp* OR customer* OR patient*)):ti,ab,kw	262
#1	(mystery NEAR/1 (client* OR shopp* OR customer* OR patient*)):ti,ab,kw	488

Table S6. Mystery client methodologies to evaluate abortion care and access

Author (year)	Jurisdiction	Theme	Aim	Methods	Setting (Who is being "shopped"?)	Outcome
Guilbert & Bois 2023a [14]	Canada	Service availability; Information quality	To identify the quality of access to medication abortion in Quebec abortion clinics	Calls	Abortion clinic staff	Wait time, number of appointments, and telemedicine availability of MA; geographic distribution of services; quality of experience rated by mystery client
Guilbert & Bois 2023b [15]	Canada	Information quality	To assess whether sufficient information is provided by abortion clinics for patients to make an informed choice between surgical and medication abortion	Calls	Abortion clinic staff	Assessment of quality and type of information provided; quality of experience rated by mystery client
Guarna et al. 2023 [16]	Canada	Service availability	To determine the proportion of pharmacies in Hamilton, Ontario, that had combination mife/miso in stock at any given time	Calls	Pharmacies	Availability of MA: in stock, willing to order, reasons why medication not in stock
Berglas et al. 2022 [27]	USA	Service availability	To explore how service disruptions in Louisiana disparately affect access to abortion care based on geography	Calls	Abortion clinic staff	Determine whether clinics were open and scheduling abortion appointments during COVID-19 pandemic, and changes in abortion rates and types related to service disruptions
Beshar et al. 2024 [28]	USA	Service availability	To examine California pharmacy mifepristone and misoprostol dispensing	Calls	Pharmacy staff	Immediate availability, availability to order, and wait time for MA from pharmacies
Bryant & Levi 2012 [29]	USA	Information quality	To identify the accuracy of medical information provided by crisis pregnancy centers in North Carolina	Calls; Visits	CPC counselling staff	Review of content and medical accuracy of information provided by the CPCs
Cartwright et al. 2018 [30]	USA	Service availability	To identify geographic locations and services of abortion facilities	Calls; Systematic online search	Abortion clinic staff	Geographic distribution of abortion clinics; availability of MA vs. procedural abortion; inter-state travel requirements
Frasik et al. 2023 [31]	USA	Information quality	To understand the practices of crisis pregnancy centers in a	Calls	CPC staff	Quality of information provided by CPC staff; record of comments that

			state with supportive abortion policies			were inflammatory, emotionally-provocative, biased, or medically-inaccurate
Kaller et al. 2021 [32]	USA	Service availability	To explore the impact of COVID-19 on abortion services	Calls; Systematic online search	Abortion clinic staff	Impacts on access to abortion during Covid-19 closures; distance to nearest open facility; telehealth uptake
Kawamoto et al. 2023 [33]	USA	Service availability	To assess access to abortion pills from California clinics providing state-funded family planning services	Calls	Abortion clinic staff	Availability rates of MA & referral to MA providing clinic
Khidir et al. 2023 [34]	USA	Service availability	To determine variation in state Medicaid agency responses to coverage questions on out-of-state abortion care	Calls	Medicaid agency staff	Coverage policies for out-of-state abortion care as represented by agency staff compared with federal policy
Mnuk et al. 2024 [35]	USA	Service availability	To identify the availability and accessibility of mifepristone and misoprostol at Oregan pharmacies	Calls	Pharmacy workers	Availability of misoprostol & mifepristone; wait time to order when not in stock or unwillingness to stock
Peart et al. 2024 [36]	USA	Service availability	To understand the demand for and access to nearest clinics offering medication abortion for North Carolina college students	Calls	Abortion clinic staff	Distance, cost, and wait times to access MA
Riley et al. 2023 [37]	USA	Service availability	To understand demand for medication abortion, and barriers to care among Washington public university students	Calls	Abortion clinic staff	Nearest abortion-providing facilities for students, and potential barriers to access
Roberts et al. 2021 [38]	USA	Service availability	To determine the impact of COVID-19 on abortion service availability, numbers, type, and timing in Louisiana and neighbouring states	Calls	Abortion clinic staff	Clinic operations, scheduling rates, wait times, and service data during early COVID-19
Vinekar et al. 2023 [39]	USA	Service availability; Information quality	To understand how state crisis pregnancy centre (CPC) policy influences access to early pregnancy confirmation at CPCs compared to abortion facilities	Calls	CPC and abortion clinic staff	Wait times, testing requirements, and costs at abortion clinics compared to CPCs

Diamond-Smith et al. 2019 [40]a	India	Information quality	To assess the quality of information provided by pharmacists selling medication abortion	Visits; Interviews	Pharmacy staff	Pharmacist self-reported knowledge of MA compared to practice dispensing MA; quality of information provided to mystery clients
Diamond-Smith et al. 2019b [41]	India	Information quality	To determine the effectiveness of an infographic in improving the quality of abortion counselling provided by pharmacists	Calls; Survey	Pharmacy staff	Changes in quality of information provided by pharmacists with infographic compared to those without; quality of experience assessed by mystery client
Dixit et al. 2025 [42]	India	Service availability; Information quality	To identify pharmacy availability, accessibility, and dispensing practices of mifepristone/misoprostol in the absence of a prescription	Visits	Pharmacy staff	Pharmacy availability of MA with/without prescription; quality of interaction with pharmacy staff/quality of experience assessed by mystery client
Percher et al. 2021 [43]	India	Information quality	To assess the influence of gender and marital status on information requested and provided by pharmacists selling medication abortion	Visits	Pharmacy workers	Quality of information provided by pharmacists about MA; variation in information provided according to presentation of mystery client; quality of experience assessed by mystery client
Powell-Jackson et al. 2015 [44]	India	Service availability; Information quality	To identify pharmacy practices offering medication abortion	Visits; Interviews	Pharmacy workers	Self-reported pharmacist knowledge of MA compared to practice dispensing; quality of information provided to mystery clients
Billings et al. 2009 [45]	Mexico	Service availability; Information quality	To determine the information provided by pharmacy staff when prompted for advice about medications for abortion, in particular misoprostol	Visits	Pharmacy staff	Availability of MA and quality of information provided by pharmacist
Clyde et al. 2013 [46]	Mexico	Information quality	To identify access to abortion information and services in Mexico City for adolescent girls	Visits; Clinic staff surveys; Client surveys,	Abortion clinic staff who provide abortion counselling and information	Quality of information provided by abortion providers; difference in counseling and information provided to adolescents with/without accompanying adult clinic; quality of

						experience assessed by mystery client
Lara et al. 2011 [47]	Mexico	Service availability; Information quality	To determine the frequency and circumstances of pharmacy vendor recommendation of misoprostol for abortion	Visits	Pharmacy workers	Information gaps in pharmacy interactions related to dispensing MA emergency contraception and other reproductive health topics
O'Neil 2017a [48]	Turkey	Service availability; Information quality	To assess the availability of abortion care at state hospitals	Calls	Hospital staff within OBGYN department	Abortion availability; quality of information provided; provider attitudes
O'Neil 2017b [49]	Turkey	Service availability	To explore the availability of abortion care at state and private hospitals in Istanbul	Calls	Hospital staff within OBGYN department	Differences in availability of abortion services in state and private hospitals; alignment of access with current law
O'Neil et al. 2024 [50]	Turkey	Service availability	To identify the availability and cost of abortion services in public and private hospitals	Calls	Hospital staff within OBGYN department	Restrictions to abortion access; cost; geographic differences in access
Huda et al. 2014 [51]	Bangladesh	Service availability; Information quality	To explore the availability and provision of misoprostol and other medications for menstrual regulation among pharmacies	Visits	Pharmacy staff	Quality of information provided by pharmacists about MA including review of complications; variation in information provided according to presentation of mystery client
Huda et al. 2018 [52]	Bangladesh	Service availability; Information quality	To understand quality and accuracy of mifepristone-misoprostol provision in urban pharmacies	Visits; Interviews	Pharmacy staff	Quality of information provided by pharmacists about MA; assessment of mystery client visits compared to self-reported survey responses
Arango et al. 2025 [53]	Colombia	Information quality	To determine whether online sellers provide the instructions and medication dosages necessary for effective medication abortions	Online/text messaging	Online MA sellers	Pharmaceutical content of MA packages purchased online; accuracy of information provided by sellers; counselling and instruction provided by online sellers
Moore et al. 2020a [54]	Colombia	Service availability; Information quality	To explore interactions between pharmacy staff and patients attempting to purchase misoprostol	Visits	Pharmacy workers	Pharmacist knowledge of MA; misinformation/recommendation of contraception for MA
Ganle et al. 2020 [55]	Ghana	Service availability	To determine stock and sale of misoprostol over-	Visits; Survey	Pharmacy workers	Availability of misoprostol; information requested by pharmacists;

			the-counter at community pharmacies			assessment of mystery client visits compared to survey responses
Gbagbo et al. 2023 [56]	Ghana	Service availability	To determine the availability of second-trimester abortion services in health facilities in Accra	Visits	HCPs who provide abortion care at various health facilities	Type of provider, methods, cost, and availability of second-trimester abortion services
Moore et al. 2020b [57]	Indonesia	Service availability; Information quality	To investigate the experiences of women attempting to purchase misoprostol online	Online messages	Online MA sellers	Pharmaceutical content of MA packages purchased online; accuracy of information provided by sellers
Moore et al. 2022 [58]	Indonesia	Service availability	To investigate the experiences of women attempting to purchase misoprostol online	Online/text messages	Online MA sellers	Landscape of MA availability online; interactions with online MA sellers; actual accessibility of purchasing MA online
Reiss et al. 2016 [59]	Kenya	Service availability; Information quality	To assess pharmacy staff knowledge and provision of abortion information and methods	Visits	Pharmacy workers	Information on MA and procedural abortion provided by pharmacists
Sigdel et al. 2022 [60]	Nepal	Service availability; Information quality	To understand medication abortion drug dispensing practices among pharmacy staff	Visits	Pharmacy workers	Variation in information provided according to presentation of mystery client and pharmacist demographics; reasons for refusal
Solheim et al. 2020 [61]	Tanzania	Service availability; Information quality	To identify how misoprostol perceived, accessed, and used off-label in Dar es Salaam	Visits	Pharmacy workers	Pharmacist knowledge of MA; misinformation/recommendation of contraception for MA
Hendrickson et al. 2016 [62]	Zambia	Service availability; Information quality	To explore client experiences seeking medication abortion from pharmacies	Visits	Pharmacy staff	Pharmacy worker behaviour and sales of MA; quality of information provided by pharmacist; quality of experience assessed by mystery client
Lara et al. 2006 [63]	Latin America	Service availability; Information quality	To examine pharmacy staff knowledge and provision practices of misoprostol and other medical abortifacients	Visits; Interviews	Pharmacy workers	Pharmacist knowledge of MA and misinformation or recommendation of contraception for MA
